# New concepts in fulminant myocarditis and risk of cardiac mortality

**DOI:** 10.18632/oncotarget.21393

**Published:** 2017-09-30

**Authors:** Enrico Ammirati, Manlio Cipriani, Paolo G. Camici

**Affiliations:** Enrico Ammirati: “De Gasperis” Cardio Center and Tranplant Center, ASST Grande Ospedale Metropolitano Niguarda, Piazza Ospedale Maggiore, Milan, Italy

**Keywords:** fulminant myocarditis, outcome, mechanical circulatory support, endomyocardial biopsy, lymphocytic myocarditis

Acute myocarditis is an inflammatory disease of the myocardium that affects mainly young people, and can present with symptoms ranging from chest pain to cardiogenic shock [1]. In 1991, Liebermann et al [2]. tried to join the clinical and pathological manifestations in a precise entity called fulminant myocarditis. Major limitation of their description of fulminant myocarditis was that it was based on 4 patients who underwent endomyocardial biopsy (EMB). They used this definition: patients that become acutely ill (within two weeks since symptoms’ onset) after a distinct viral prodrome, with severe cardiovascular compromise, ventricular dysfunction and multiple inflammatory infiltrates of lymphocytes and macrophages by histologic study. They included only patients with lymphocytic myocarditis, excluding patients with other inflammatory infiltrates, such as eosinophilic myocarditis or giant cell myocarditis that can often present with a fulminant course, and are often clinically undistinguishable [3].

In 2000, McCarthy et al [4]. published a retrospective series of 15 cases of fulminant myocarditis compared with patients with acute myocarditis with reduced left ventricular ejection fraction (LVEF). They followed the above-mentioned definition of fulminant myocarditis including only lymphocytic forms who underwent EMB between 1984 and 1997. They did not include autoptic cases, and they included patients with a previous fulminant myocarditis with symptoms’ onset up to 12 months. All patients had a LVEF below 40%, and surprisingly patients with fulminant myocarditis had a normal cardiac output (5 L/min as mean value). Among 15 patients with fulminant myocarditis only 1 patient died with a reported 93% survival. On the contrary, patients with acute myocarditis and HF symptoms without fulminant presentation had a worse outcome with a 45% survival free from heart transplantation (HTx) at 11 years. In 2005, Asaumi et al [5]. described a series of 14 patients with fulminant myocarditis from 1996 and 2001 treated with percutaneous extracorporeal membrane oxygenator (ECMO) that is a type of mechanical circulatory support (MCS). They found that ECMO could be useful to increase survival in these patients with fulminant myocarditis and they observed a 71% in-hospital survival. The relevance of this study was that it demonstrated the advantage of using ECMO in refractory fulminant myocarditis.

In a recent review Ginsberg put forward a new definition of fulminant myocarditis as patients with a distinct onset of symptoms in the prior 2 weeks, with severe symptoms of HF (New York Heart Association [NYHA] class IV) and hypotension or overt cardiogeninc shock needing inotropes, vasopressor and/or MCS [6]. They also differentiate this clinical scenario, from patients with severe acute myocarditis, as patients with indistinct onset of symptoms, HF (NYHA III-IV) but without hypotension. Thus, they moved from a clinic-pathologic entity [2] to a practical clinical scenario for physician.

In our recent study, published in Circulation on August 8^th^, 2017 [7], we described the largest group of patients with fulminant myocarditis (*n* = 55, Table 1) compared with patients with acute myocarditis enrolled at the Niguarda Hospital in Milan and San Matteo Hospital in Pavia (both in Italy) from 2001 to 2016. We believed that McCarthy et al. could have underestimated the real mortality rate potentially excluding those patients with a rapid unfavorable course that were not referred to Johns Hopkinson Hospital as they that had died before performing an EMB. Our key enrolment criterion was recent onset of symptoms (within 30 days from hospital admission or within 2 weeks in the sub-analysis of adults with post-viral myocarditis that excluded eosinophilic, giant cell and sarcoid), thus capturing acute inflammatory myocardial injury close to the time of its onset. In our study, we found that in the whole population (*n* = 187), the rate of in-hospital death or HTx was 25.5% versus 0% in fulminant myocarditis compared with patients with non-fulminant acute myocarditis (*p* < 0.0001). In the subgroup analysis that included 130 adult patients with acute post-viral myocarditis, in-hospital mortality was 11.8% in the fulminant group compared with 0% (*p* < 0.0001) in the post-viral non-fulminant acute myocarditis. Kaplan-Meier curves of HTx-free survival showed worse outcome in the fulminant compared with non-fulminant group at 9 years of follow-up (80.7% versus 100%, respectively; log-rank *P* < 0.0001). Another important finding was that patients with lymphocytic post-viral fulminant myocarditis with available histology (*n* = 25) generally had more inflammatory infiltrate at histology compared with patients with lymphocytic post-viral non-fulminant myocarditis (*n* = 5). Adult patients (*n* = 30) with lymphocytic myocarditis who received MCS or died during hospitalization had a greater degree of inflammatory infiltrate (defined as 3A–4 or 2R–3R, 55.6%) compared with patients who survived without MCS (8.3%; *p* = 0.018). For the first time, we provided histo-pathological scores based on the extent of immune cell infiltration and necrosis borrowed from the 1990 and 2004 International Society for Heart and Lung Transplantation (ISHLT) heart rejection grading system [8]. This scoring system can be a useful tool to compare the degree of inflammatory infiltration in patients with acute myocarditis. Finally, despite greater improvement in LVEF was observed during hospitalization in fulminant compared with non-fulminant forms, the proportion of patients with LVEF <55% at last follow-up was higher in patients with fulminant myocarditis.

**Table 1:** Proposed new operative definition of fulminant myocarditis

SUGGESTED DEFINITION OF FULMINANT MYOCARDITIS:
1. Patients become acutely ill (<1 month since symptoms’ onset)
2. Hemodynamic instability due to cardiogenic shock or arrhythmias (including sudden death)
3. Need for hemodynamic support (inotrope/mechanical circulatory support)
4. Multiple foci of active myocarditis by histologic study

In the conclusion, we report the words used by the associate editor of Circulation, Dr. Nancy Sweitzer who handled the manuscript (from “Circulation on the Run”, podcast, August 8^th^, 2017): “this was a very important paper because it really looked inclusively at myocarditis in the modern era, and showed us where perhaps bias in prior studies had led us astray in terms of our beliefs about, particularly the outcomes in this syndrome.”
